# Mr. Sheldrake, in Answer to Mr. Watt

**Published:** 1800-09

**Authors:** T. Sheldrake

**Affiliations:** No. 50, Strand


### *To the Editors of the Medical and Phyſical Journal.*

GENTLEMEN,

AS the firſt object of your valuable Publication is to diſſeminate the knowledge of uſeful diſcoveries, ſo, I preſume, the next, in order as in utility, will be to prevent the propagation of error; and if, in execution of the firſt part of your plan, you ſhould accidentally promulgate any thing, under the idea of its being an uſeful diſcovery, though, in fact, it was previouſly well known, and proved by experience, to be inadequate to what it profeſſes, I apprehend you will not be unwilling to admit ſuch obſervations as may tend to reduce it to its juſt value in the eſtimation of your readers.

The conſideration of theſe circumſtances, which, I preſume, regulate the conduct of your Publication, has induced me to trouble you with the following obſervations on a Communication from Mr. R. WATT, in your laſt Number, which he calls, Deſcription of a New Machine for curing diſtorted Limbs. My intention is to ſhow, firſt, that it is not a new diſcovery; and, ſecondly, that although it may be uſeful on ſome occaſions, it is abſolutely incompetent to effect all that he ſo unreſervedly attributes to it.

In doing this, I have no intention to impeach Mr. Watt's veracity, or the purity of thoſe motives which, he ſays, have induced him to communicate his diſcovery to you; for it may be granted, that he has known nothing more on the ſubject than what has emanated from his own mind; it may be granted too, that he has cured by this inſtrument, ſome deformity that he calls varus, or valgus; and yet, all that I mean to advance in abatement of his claim to the originality or utility of his invention, may be ſtrictly true.

When profeſſional men, in retired ſituations, are induced to turn their minds to particular ſubjects, without previous information, they conclude, and juſtly too, that whatever ariſes in their minds, is *their own invention*; — but, when they introduce their diſcoveries to the world at large, they will often find, and perhaps not without chagrin, that what was new to them, had been previouſly long and well known to many; that, what in their limitted experience ſeemed to be advantageous, had been found ineffectual in the hands of many others; and, they will gain this advantage by this diſcovery, viz. to be more guarded in their aſſertions reſpecting their own imaginary inventions, and more cautious in aſſigning unworthy motives to thoſe who may not act exactly as they have thought proper proper to do. This I believe to be Mr. Watt's ſituation, for the following reaſons:

Firſt, When I was in Dublin, in the year 1796, a gentleman employed me to cure his child's diſtorted foot. Upon enquiring what, if any thing, had been done previous to my ſeeing the caſe, I was told the late Mr. Deaſe had been conſulted, and he adviſed the parents to ſend for a pair of the well-known Edinburgh boots: he deſcribed theſe as being intended to preſs and compreſs the feet till they were reduced to the natural form. I had no communication with Mr. Deaſe on the ſubject; but as he deſcribed the inſtrument as being well known in Edinburgh, and attributed the ſame powers and mode of operation to it as Mr. Watt does to his *invention*, I have ſome reaſon to conclude they are not very diſſimilar.

Secondly, Dr. Aitkin, in his Eſſays on Fractures, &c. deſcribes an inſtrument for the ſame purpoſe, and which is to act in the ſame manner upon the diſtorted feet of children; he gives a plate of this inſtrument, which certainly reſembles that of Mr. Watt's in part. In deſcribing this plate, he ſays, the machine much reſembles the boot of Hildanus. Here is both ancient and modern information, which have moſt unaccountably eſcaped that ſpirit of reſearch, which induced Mr. Watt to turn over " every volume of ſurgery he could lay his hand on, without finding any thing to his purpoſe."

Thirdly, It is at leaſt fifty years ſince a ſhoe-maker, whoſe name was Holmes, made known, as his invention, a method of curing the diſtorted feet of children: It conſiſted in binding them firmly, and then inſerting them in a pair of boots or ſhoes, that are connected together by means of a ratchet and ſpring. His intention was, like that of Mr. Watt, to preſs upon the convex ſide, and then twiſt and turn the feet out; and we ſee the means he uſed are the ſame. As this man practiſed his art for the reſt of his life, and a perſon is ſtill living who continues to do ſo, their long experience may prove more than Mr. Watt's; it is well known that they have *imperfectly cured* ſome very ſlight caſes, but they never were able to cure completely any one important caſe of diſtortion.

Having ſaid thus much on the *originality* of Mr. Watt's invention, it remains to conſider its *efficacy*; for, after all that can be ſaid, it is of little conſequence whether a man makes a diſcovery, borrows the deſign of another, without leave or acknowledgment, revives an obſolete or gives publicity to an obſcure invention, provided that ſociety at large is benefited by his communication; Mr. Watt ſays the individual is ſure of being rewarded as he deſerves. It is to be wiſhed that the terms varus and valgus, as applied to theſe deformities, were baniſhed banifhed from our Vocabulary; for as they only defcribe the pofition of the foot, without indicating the ftate of the difeafed parts, and are applied indifcriminately to all, they only ferve to confound inftead of illuftrating the fubject. Even Mr. Watt *seems* to have but very crude ideas of the facts of this difeafe; he fays, "THIS DISORDER (i. e. the two difeafes called varus and valgus) may be occafioned by different caufes; by a morbid ftate of the bones, or contraction of the mufcles; by original mal-conformation, or wrong pofition in the womb. *But from whatever caufe the diftortion may proceed, the reducing the bones to their natural pofition muft be accomplifhed by nearly the fame means; that is, by judicious preffure upon the convex side. In fome* cafes the diforder lies in the ancle joint, while the leg and knee are perfectly natural; in others, *and perhaps the greateft number, it is occafioned by a bending of the bones of the leg*, by which the toes are turned either out or in, according as the bones are bent to the one fide or the other."

I fhould be forry to mifreprefent, though the obtufenefs of my intellects may oblige me to mifunderftand, Mr. Watt's meaning: If I do underftand it, it is this; that when the firft cafe was put under his care *he had never feen fuch a cafe treated before*; he immediately began to turn over every volume of Surgery he could lay his hands on; but finding nothing to his purpofe, he BEGAN to think for himfelf, and, *fortunately*, fell upon the following contrivance, which, he is happy to fay, has anfwered his purpofe extremely well.

From this it is evident, that all his actual knowledge of the difeafe, its caufes, confequences, and method of cure, was derived from this *fingle cafe*; for the fecond cafe he relates, came to his hands after his communication to you was written and ready to be fent off; of courfe, is only to be confidered (if I may adopt a parliamentary phrafe) as a rider placed upon the back of the original bill, in hopes it might add to the confequence of that, though itfelf had no pretenfions to any importance at all. And, confidering he has built all his fuperftructure upon this flender foundation, it is not very furprizing that he fhould fometimes be miftaken, often inaccurate, and not unfrequently obfcure.

I have been occupied upon this fubject almoft twenty years, during that time have feen feveral hundred cafes, including, perhaps, all the varieties of the difeafe, and am warranted in faying, I have never feen more than one cafe where the feet were turned inwards, and the bones of the legs *bent*; on the contrary, in all thofe cafes except the one I allude to, the bones of the legs were ftraight, in moft of them preternaturally fo.

As

As I have fully explained this fact in another place,* I content myfelf with afferting here that it is fo, and hope your profeffional readers will examine more than one or two cafes, before they regulate their opinions by the dictum of Mr. Watt.

Mr. W. fays, "From whatever caufe the diftortion may proceed, the reducing *the bones*" (i. e. as I fuppofe, the parts concerned in thofe diftortions) "to the natural pofition muft be accomplifhed by nearly the fame means, viz. by judicious preffure upon the convex fide."

It is to be obferved, that in thofe difeafes where the feet turn inwards, and which, I prefume, Mr. W. will call valgus, the deformity is occafioned by deranged combination of the bones of the foot, though in fome the legs may be bent: that where the feet turn out, and I fuppofe thefe deformities are to be called varus, there is more frequently diftortion of the bones of the leg, and the feet are frequently fo preffed againft the fibula while in utero, as to make the feet as flat as the hand of an adult; it is difficult to believe the fame mode of treatment will cure both. If I may venture to add my mite to the intelligence of Mr. Watt, I fhould fay, there is, in every cafe, one condition of the bones that will require peculiar treatment; there is another condition of the ligaments, and a third of the mufcles; that each require treatment peculiarly adapted to them; and I can fafely aver, that of more than an hundred cafes which I have cured perfectly, no two were exactly alike, or could have been cured without adapting the mode of treatment peculiarly to each. If, after confidering this fact, Mr. Watt will continue to fay that all thefe difeafes may be perfectly cured by being fqueezed into the boxes he has defcribed, I believe few intelligent practitioners will fupport his opinion. As all that Mr. Watt fays, though *he is inclined to think there is no kind of diftorted limb, however formidable it may appear, but may be cured by the fame means*, depends upon what has been done in two (no doubt very flight) cafes, and he has given *general accounts* of the difeafes to which he propofes to apply the fame mode of treatment, and which accounts are not very correct or juft, it will be needlefs wafte of time to follow him through the defcription of his plan; one particular, however, is too remarkable to pafs unnoticed; he fays, "*To keep the child quiet while we were putting on the inftrument, and through the night, I found it neceffary to ufe a gentle opiate once in twenty-four hours. This, however, did no hurt to the child's health; and as foon as the operation was finifhed, we gave it up entirely!*"

Mr.

* Practical Effay on the Club-foot.

Mr. Watt has faid the cure was done with the greateft *eafe*; now, if this was fo, furely the regular ufe of opiates was unneceffary; if, on the contrary, as I have no doubt was the fact, Mr. Watt's fqueezing fyftem was fo uneafy to the child, that he could not be kept quiet while the inftrument was putting on, or obtain a fingle night's reft *without a gentle opiate once in twenty-four hours*, it was natural for Mr. W. to keep his patient under the influence of narcotics during the whole time he was performing the cure. The medical reader will accept this as a *ftrong proof of the eafe of Mr. Watt's operation*; and it may induce him to entertain fome doubts, even if there were no ftronger reafons to doubt it, of the propriety of adopting a plan that includes *the neceffity of keeping even an infant under the influence of* foporific drugs during the whole time of performing a cure.

Having fhewn what he has done, Mr. Watt proceeds to fhew what he thinks *may be done by his invention*; and as there are ftrong reafons for not mifreprefenting what he has faid on this fubject, I truft I fhall be excufed for making the following extract from his defcription, &c.

He fays, "*When I firft thought of this inftrument*, I had no idea of its being applied to any other cafe but the vari and valgi, and thefe only when the diforder happened to lie in the ancle joint. *But I have fince thought*, that by a fmall alteration, it may be applied to all the different kinds of diftorted limbs with confiderable eafe and advantage.

"Fig. 2, reprefents the half of the machine, in every refpect fimilar to the one above defcribed, fave only, that one of its fides from the heel up is removed, *and in place of it a fpring, the under end of which is fixed at* E, *to the fide of the heel*; *the other, by means of the ftrap* F, *to the top of the machine. The convex fide of the fpring, ftuffed and covered with foft leather, being applied to the convex fide of the limb, by means of the ftrap* F, *any degree of preffure can be made, which the practitioner may judge neceffary.* The fide of the leg of the machine, oppofite the fpring, muft have a head as reprefented at G, to reft againft the outer or inner condyle of the femur, according as the cafe may require. *If the bones are bent forward, as is often the cafe, by fixing the fpring upon the fore fide, and caufing it to prefs upon the convexity of the limb, they may be cured in the fame manner as above directed.* By ufing a fpring inftead of ftraps round the limb, as directed by Mr. Benjamin Bell, a more uniform and effectual preffure can be made. *With regard to the ftrength of the fpring, no general rule can be given; this muft depend entirely upon the age of the patient, or the degree of force required to ftraighten the limb.*"

Thus

Thus far Mr. Watt. Will you excufe me, gentlemen, for informing you, that I have been employed many years upon this fubject; having confidered this clafs of difeafes in a light they had never been placed in by any perfon whatever; having, by reiterated experiments, difcovered and perfected a method of curing them much more effectual than any that had previoufly been known, I obtained a patent to fecure the benefit of my difcovery to myfelf for the ufual term: In doing this, however, I did not incur Mr. Watt's cenfure by "concealing, as far as poffible, my difcovery from the public;" on the contrary, I refolved to make it as public as poffible, from a conviction that by fo doing I fhould extend its utility, and, of courfe, my own reputation.

In 1798, I publifhed a Practical Effay on the Club-foot, &c. with a view to afcertain how far they were curable; and in that Effay I inferted a correct copy of the Specification of my Patent, with plates engraved from the drawings which accompanied it. As that Effay has acquired a character which it would not become me to defcribe, I fhall content myfelf with making an extract from the fpecification contained in it, which extract relates to the fubject in queftion. (*Practical Essay on Club-Foot*, p. 198.

"I fhall illuftrate the method of treating thofe diftortions or deformities which arife from the improper form of bones, by explaining the treatment of curvature in bones of the leg, which is one of the moft frequent difeafes of that defcription; *whether the bones bend inwards, outwards, or forwards, is of no consequence, as the principle on which the remedy is to be applied is the same in all.* Figures 1 and 2, hereunto annexed, reprefent a child's leg, bending outwards; the lines marked with A, B, C, in both figures, reprefent the curved fpring, intended to correct this deformity. It is evident, that if this fpring is, by bandage or otherwife, at *a*, *d*, and *c*, *e*, in fig. 1, brought into contact with the leg, the infide of the knee, as at *d*, in figure 1, and *a*, in figure 2, and bottom of the leg, which correfpond with the ends of the fpring, will form refting points for the fpring to act *from*; while its re-action, by producing preffure on the projecting part of the curve of the leg, reduces the bone towards its natural ftate. The fame effect will be produced by either of thefe methods, the difference between the modes of producing the effect being, that in figure 1, the bandages at *a*, *d*, and *c*, *e*, form the refting points, and preffure is made upon the curve of the bone by the fpring at *b*. But in figure 2, the ends of the fpring, *a* and *c*, form the refting points, and the re-action of the fpring produces preffure from the bandage, *b*, *d*, upon the curve of the bone."

The resemblance between Mr. Watt's invention and mine is very evident, but the priority is indisputably with me.—Mr. W. may perhaps say, he has never seen my book; and, considering the distance of his residence from the metropolis, he may, perhaps, say so with truth: But it is to be remarked, that before the publication of my book, viz. in Sept. 1797, the proprietors of the Monthly Magazine inserted a detailed account of my Specification, with the explanatory plates, in their publication, which, from its very extensive circulation, it is most probable did fall into Mr. Watt's hands; though its contents made so little impression on his mind, that, at a subsequent period, he actually mistook the part I have quoted for the offspring of his own brain.

After saying thus much upon the resemblance of Mr. Watt's invention to mine, it is necessary to remark the difference between the inventors. Having by a series of experiments, followed for a length of time, which it would seem like affectation to dwell upon, discovered a new and effectual method of curing these diseases, I then made it public, with proofs that have incontestibly established its superior efficacy; but the effects of a long and successful practice, do not authorize me to assert, in the language of Mr. Watt, that "*there is no kind of distorted limb, however formidable it may appear*," but may be cured by the same means that have been successful in those cases that I have already cured. Mr. Watt, on the contrary, is accidentally called to a *single case*, which he knows not how to treat; he, naturally enough, began to turn over every book of Surgery within his reach, in hopes of gaining information, but without success; he then sets his own invention to work, and discovers *something* that proves successful; he immediately becomes omniscient on the subject, applies his invention, *in imagination only*, to numberless diseases of various kinds, and determines that all distorted limbs, however formidable they *may appear*, may be cured by the same means. Whence he derives his confidence, cannot be known; it certainly could not be from his knowledge and experience, as he had never seen but one case before he wrote the paper he has communicated to you, and one more before he sent it. This, to some of your readers, may appear absurd; it is not all the absurdity, however, contained in Mr. Watt's communication: For example, in his exordium he says, "Surgery, *so far as he knows*, has made little or no provision to cure the distorted limbs of infants;" and, *immediately afterwards*, he says, "though many have in other places remained, from whatever cause, in that situation for life; yet, *in this part of the country*, *I have not seen a single instance of one who has not been cured of the disease.*

It

It ſeems that Mr. Watt is not willing to raiſe the credit of his own country above its proper level; for if he was inclined to do it juſtice, and if the latter part of his aſſertion be true, he ſhould have ſaid, Surgery, in that part of the country, has done a great deal in the cure of theſe diſeaſes.

As Mr. Watt juſtly cenſures the impropriety of reſorting to itinerants, quacks, and *pretenders*, upon ſuch ſubjects, and expoſes the falſhood of the opinion held by ſome, that regular ſurgeons can do nothing for the cure of theſe diſtortions, it is not a little ſurpriſing that when Mr. W. was called to the caſe of Wardrop, he had no knowledge of the proper method of treating it; it is *more* ſurprizing that notwithſtanding the diſeaſe had been extirpated root and branch from all the ſurrounding country, (undoubtedly by the exertions of regular practitioners) no friendly practitioner was to be found in the neighbourhood, to inform him by what means the diſeaſe had been ſo perfectly and univerſally cured. This argument might be extended to greater lengths than I chuſe to puſh it; for the train of ideas that Mr. W. indulges himſelf in, is too obvious to be miſtaken by the moſt ſuperficial reader.

Mr. Watt has laid much ſtreſs upon the ſaving clauſe, which he expreſſes by the words "*if taken in time*," with which he qualifies all his curative aſſertions; it is to be regretted that he has not afforded ſome criterion, by which the leſs experienced practitioner might have a chance to determine à priori, when it is not too late to undertake a cure of this kind with a probability of ſucceſs. As he has not done this, however correct the ideas may be which he annexes to thoſe words, they will afford a convenient ſubterfuge to palliate the want of ſucceſs in every caſe; thus, if any perſon ſhould attempt to practiſe that which Mr. Watt has only *thought muſt be univerſally ſucceſsful*, IF TAKEN IN TIME, and finds he does not ſucceed, the failure muſt not be attributed to want of efficacy in the means employed, or want of knowledge and experience in the operator, but merely to the circumſtance of the patient not having applied till it was too late to cure him; and thus, according to this ſyſtem of logic, the method of cure may be good, if not a ſingle patient ſhould ever be cured by it.

As few intelligent men will believe, that Mr. Watt's ſyſtem of ſqueezing the diſtorted feet of children into tin or leather boxes, &c. will be more ſucceſsful than it has been, though attempted and recommended in various ways for many years paſt, that part of the ſubject may be left to its fate: But as the other part of his INVENTION, which he has only *thought* muſt be ſucceſsful if applied to all the different kinds of diſtorted limbs, &c. ſo much reſembles the deſcription of my invention, which was inserted with explanatory plates in the Monthly Magazine, that I ſhall believe it was copied therefrom, until there is ſtrong proof to the contrary, may I be permitted to mention what my experience authoriſes me to ſay upon what Mr. Watt *conjectures* may be the conſequence of applying *his invention* in ſuch caſes? It is this; if any perſon ſhould apply the inſtrument deſcribed by Mr. Watt, to ſuch caſes as he there recommends it for, he will find it *perfectly uſeleſs*, becauſe he will find it will not be poſſible to keep it ſo long in its proper ſituation as to produce any effect whatever.

I am to apologize for having obtruded longer upon your patience than I intended; but, I truſt you are ſenſible that there is no circumſtance more certain of producing miſchief in any part of the healing art, than that of obtruding crude notions of any diſeaſe, and recommending modes of curing that diſeaſe as certain of being ſucceſsful, though only founded upon ſome trifling experiment, and hope you may think the time not unuſefully employed, that has been occupied in placing the importance and originality of Mr. Watt's communication in its juſt point of view.

Though a reader of your Journal from its commencement, I did not ſuppoſe this ſubject would have found a place in it; I now entertain a different opinion; and if you ſhould think it deſerving your attention, will trouble you with an account of ſome facts that have not been generally thought poſſible; among others I ſhall endeavour to aſcertain at what period it is not too late to undertake the cure of diſtortions in the feet, &c. I ſhall prove that perſons born with ſuch defects, have been perfectly cured at the age of nine, ten, or eleven years; and I ſhall infer from thence, that many caſes of this kind, which have long been abandoned as incurable, may be perfectly reſtored to their natural ſtate: This, if you approve of it, will be the ſubject of my next communication. I am,

Gentlemen,

Your moſt obedient ſervant,

*No. 50, Strand.* T. SHELDRAKE.

STATE

